# Adolescence

**Published:** 1909-04-10

**Authors:** 


### Diseases of Children.

### ADOLESCENCE.

The age of adolescence, from twelve to eighteen years, hardly receives sufficient attention in medical literature; and yet it is in some respects of peculiar importance. It is the age of accelerated physical and psychical development, by leaps and bounds rather than a steady progression, varying greatly in different children. The growth of bones and muscles is rapid. In girls it is most marked at 12-15 years; in boys at 14-17 years of age, and it takes place rather earlier in large than in small children.

Important functions arise and develop. Sex asserts itself. At this period the development of normal periodicity in girls is of the utmost importance, and should take precedence of that of muscle and mind. The frame is preparing for maternity. The voice changes. Vaso-motor instability is great, and exhibits itself in blushing, orthostatic albuminuria, fainting, etc. Sensations of taste and smell are more epicurean and psychical. Individualities become differentiated. Sentiment, self-consciousness, love, religious feeling and ambition arise. It is the age of hopes, ideals, and tender sentiments. There is a craving for knowledge of body and mind, and a delight in rhythm, music, and singing. Yet the ideas are inchoate, the mind lacking in precision and conscious power, and there is a liability to exaggeration and excess, and the feelings fluctuate rapidly in character. Even now the general tendency is objective rather than subjective, and training should be manual, gymnastic, in sports and games, industrial as well as mental and moral.

At this age the liability to the diseases of childhood is much reduced, and diseased processes take on the adult type. Those which are peculiar to this period depend upon the arrest, defect, or excessive development of some organ or function. Hence we find hooliganism, perversions, juvenile crime, and secret vice. Many of the so-called disorders of adolescence are due to remedial causes, and are really independent of age, except in so far as the age modifies the mode of life and education. Thus we can ascribe to insufficient sleep, defective hygiene, the bad print of school books, unsatisfactory diet, and examination strain, such minor affections as anorexia, dyspepsia, constipation, headache, anæmia, visual defects, epistaxis, and nervousness. Spinal curvature, most common at six to fourteen years of age, but occurring at fourteen to seventeen years of age in 5 per cent. of the cases, may be due to the above conditions, bad positions at work, unsuitable desks and seats, and the debility consequent on too rapid growth.

The diet should be very liberal at this age. It is simply astonishing what large amounts these children eat with comfort and impunity. A liberal supply of calcium is needed for the rapidly growing bones, of iron for the blood, phosphorus for the brain, protein for muscle, carbohydrates for exercise, fat for heat, and oxygen for metabolism. Let those in authority at boarding schools see that such needs are amply provided for. Slight cardiac failure, usually of short duration, may result because the growth of the body is more rapid than that of the heart, which nearly doubles its size at puberty. Hence arise languor, feeble pulse, shortness of breath, and palpitations. Old cardiac mischief, notably any adhesion of the pericardium, is apt to lead to signs of cardiac failure. Cardiac arrhythmia may depend on insufficiency of muscle or on nervous causes. Nervous palpitations are not uncommon in girls for a few weeks or months before the first menstruation, in conjunction with anorexia, dyspepsia, constipation, inertia, irritability, and sleeplessness. Tall, rapidly growing, thin, non-anæmic girls at this period may suffer from a sense of fulness and shortness of breath, and exhibit hypertrophy of the left ventricle. Others may become chlorotic.

So-called hysterical symptoms exhibited at this period of life include abnormal eating, fasting, capricious appetite, pica, barking cough, and stuttering. Excessive dreaming and sleep walking are not uncommon. Migraine occurs in girls. Sometimes there are troublesome disorders of menstruation. Menorrhagia may occur at puberty, rarely at the first period. It may cause profound anæmia, and has been known to prove fatal. It should be treated with calcium lactate for three days twice a month, drachm doses of ergot for the bleeding, and bromides. Irregularity at the onset is quite common and unimportant. It must not be forgotten that an imperforate hymen or other malformation may not give evidence of its presence until the onset of the catamenia.

Mental and nervous disorders are common in the form of emotional disturbances, hysteria, hysterical fits, and less frequent as religious melancholy, hallucinations, ecstasy, trances, preaching mania, suicidal tendencies, horror of death, hystero-epilepsy, a proclivity to epilepsy, delusions, malingering, neurasthenia, and sexual psychoses. Hysteria is often due to a sexual shock which is brooded over in secret, and can be cured by talking about it. All these symptoms are more likely to develop in defectives, but normal children may first be found defective at puberty and unable to profit by teaching. General perturbations, the result of erotic excitement, may become dangerous at this age, and make it necessary to send previously harmless imbeciles to asylums. Vicious habits may be unduly developed by bad companions, imitation, and insufficient supervision in the organisation of school life. Excess depends greatly on inherited instability of the nervous system. The normal psychic features of puberty are most interesting to watch. The birth of imagination leads to reverie, inner absorption, musing, brooding, and even illusions. The vivid ideas appear as realities to some imaginative children. The critical faculty is defective, yet there arise a consciousness of self, oversensitiveness, and self-criticism. This again leads to over-assertion of the individuality and the bumptiousness of youth. The vocabulary is enlarged and words have a more definite meaning. It is the age of imitation, of folly, of dramatic roles and poses. Fortunate is the child who is under judicious guidance, for the future may be made or marred.

